# *Yadanziolide A* Inhibits Proliferation and Induces Apoptosis of Hepatocellular Carcinoma via JAK-STAT Pathway: A Preclinical Study

**DOI:** 10.3390/biology13070528

**Published:** 2024-07-16

**Authors:** Lili Lin, Qi Chen

**Affiliations:** Fujian Key Laboratory of Innate Immune Biology, Biomedical Research Center of South China, College of Life Science, Fujian Normal University Qishan Campus, College Town, Fuzhou 350117, China; qbx20180135@yjs.fjnu.edu.cn

**Keywords:** hepatocellular carcinoma, *Yadanziolide A*, apoptosis, proliferation, the JAK-STAT pathway

## Abstract

**Simple Summary:**

Liver cancer remains a serious health issue worldwide, necessitating the development of novel treatments. *Yadanziolide A (Y-A)* has shown potential in treating liver cancer. Our research evaluated *Y-A’*s effects on liver cancer cells and in a mouse model. We discovered that *Y-A* not only kills liver cancer cells but also prevents their spread and induces programmed cell death at effective doses. In animal models, *Y-A* reduced tumor growth and improved health markers. Our study also identified that *Y-A* targets specific pathways related to cancer cell survival and growth, offering insights into its mechanism. These findings support *Y-A* as a promising candidate for liver cancer therapy, highlighting its effectiveness and potential clinical application.

**Abstract:**

Liver cancer is a significant global health concern, prompting the search for innovative therapeutic solutions. *Yadanziolide A* (*Y-A*), a natural derivative of Brucea javanica, has emerged as a promising candidate for cancer treatment; however, its efficacy and underlying mechanisms in liver cancer remain incompletely understood. In this study, we conducted a comprehensive evaluation of *Y-A’*s effects on liver cancer cells using a range of in vitro assays and an orthotopic liver cancer mouse model. Our findings reveal that *Y-A* exerts dose-dependent cytotoxic effects on liver cancer cells, significantly inhibiting proliferation, migration, and invasion at concentrations ≥ 0.1 μM. Furthermore, *Y-A* induces apoptosis, as evidenced by increased apoptotic cell populations and apoptosome formation. In vivo studies confirm that *Y-A* inhibits tumor growth and reduces liver damage in mouse models. Mechanistically, *Y-A* targets the TNF-α/STAT3 pathway, inhibiting STAT3 and JAK2 phosphorylation, thereby activating apoptotic pathways and suppressing tumor cell growth. These results suggest that *Y-A* has promising anticancer activity and potential utility in liver cancer therapy.

## 1. Introduction

Hepatocellular carcinoma (HCC) is the most prevalent primary liver tumor and a leading type of liver cancer, with a high mortality rate [1,2,3]. It ranks as the third leading cause of cancer-related deaths globally, following lung and colorectal cancers. Therefore, early detection, accurate diagnosis, and the development of new and effective treatment strategies are crucial for improving patient prognosis.

Traditional Chinese Medicine (TCM) has a long history of use in liver cancer treatment [4]. However, the lack of rigorous scientific evaluation has hindered the standardization and modernization of TCM, despite its ability to prevent and delay HCC progression [5,6]. Recently, there has been a surge in pharmacological research on TCMs and their active constituents to reveal their antineoplastic mechanisms. These studies have provided valuable insights into the pharmacological activities of TCMs and their potential to combat cancer, expanding the therapeutic options for HCC.

The dried and mature fruit of the bitter wood family plant *Brucea javanica*, primarily produced in southern China [7], has been used for centuries in TCM to treat various ailments, including malaria, dysentery, and cancer, and has gained popularity as a complementary therapy for cancer patients [8,9,10].

*Brucea javanica* oil emulsion (BJOE) not only inhibits the proliferation of HepG2 and Hep3B cells but also significantly reduces tumor size and extends the survival period of H22 tumor-bearing mice [11]. Additionally, BJOE as an adjuvant to transarterial chemoembolization (TACE) is considered a potential therapeutic option for liver cancer patients. It helps improve treatment outcomes, enhances quality of life, and potentially reduces adverse effects [12,13]. However, its clinical application is limited due to low bioavailability and the complexity of its components.

*Yadanziolide A (Y-A)*, a compound extracted and purified from *Brucea javanica*, offers significant therapeutic potential in cancer [14,15]. However, as an active component of *Brucea javanica* extract, studies on *Y-A* in tumors are extremely limited. Despite these promising findings, the specific anti-tumor effects and underlying mechanisms of Y-A on liver cancer have not been elucidated.

Therefore, this research aims to fill this gap by comprehensively evaluating the effects of *Y-A* on liver cancer cells and elucidating its underlying mechanisms. By addressing the current limitations in the understanding of *Y-A’*s effects on liver cancer, this study aims to contribute valuable insights into its potential as a novel therapeutic agent for HCC.

## 2. Materials and Methods

### 2.1. Materials and Cell Culture

*Yadanziolide A* (CAS 95258-15-4, purity ≥ 98%) was obtained from Shanghai Tauto Biotech Co., Ltd. The chemical was dissolved in dimethyl sulfoxide (DMSO) supplied by Sigma (USA) and stored at −20 °C to maintain stability.

DMEM containing 10% FBS (Gibco, Life Technologies, Canberra, Australia) and 1% penicillin-streptomycin solution was used to culture HepG2, Huh-7, LM-3, and Hepa1-6 cell lines (ATCC). The HL-7702 cell line was obtained from Dr. Xiao Nanyang. The HepG2 cell line was provided by Prof. Dr. Li Daliang. The Huh-7, LM3, and hepa1-6 cell lines were obtained from Dr. Liu Yixuan.

### 2.2. Animals and Tumor Mouse Models

In the Animal Center of Fujian Normal University, male C57BL/6 mice were bred and maintained under pathogen-free conditions. All research and animal care procedures were approved by the Animal Ethical and Welfare Committee of Fujian Normal University (IACUC-20230041).

In order to establish the mouse model, a total of 2 × 10^6^ Hepa1-6 cells in 100 μL of serum-free medium were administered via injection into the mouse liver (*n* ≥ 5). *Yadanziolide A (Y-A)* has a maximum solubility of 100 mg/mL (234.52 mM) in DMSO. For experiments, a stock solution of *Y-A* at a concentration of 100 μM in DMSO was prepared. This stock solution was then diluted with PBS to the required concentration for intraperitoneal injections in the mice. Subsequently, the mice were subjected to intraperitoneal injections of either PBS or *Y-A* (2 mg/kg/day) for a duration of 14 days. Tumor growth was assessed utilizing an in vivo imaging system, followed by sacrifice of the mice to collect serum samples. Hematoxylin H&E staining was then conducted on the tumor tissues for further analysis.

### 2.3. Determination of Cytotoxicity of Y-A to Cancer Cells

To test *Y-A* cytotoxicity, LM-3, Huh-7, and HepG2 cells were seeded in 96-well plates at 2 × 10^3^ cells per well and treated with *Y-A* at concentrations from 0 to 10,000 nM for 24 h. Cell viability was then assessed using CCK-8 assay by measuring absorbance at 450 nm after incubating with CCK-8 reagent for two hours.

### 2.4. Western Blotting

Cells were harvested and extracted using cell lysis buffer solution with protease and phosphatase inhibitors. After centrifugation, protein concentration was determined and samples were prepared for immunoblotting. The PVDF membranes were blocked with 5% non-fat milk in TBS with 0.1% Tween for one hour at room temperature, then incubated with specified antibodies. Immunostaining was carried out using various antibodies from Cell Signaling Technologies and secondary antibodies—Phospho-STAT3 antibody (1:1000 dilution), STAT3 antibody (1:1000 dilution), Phospho-JAK2 antibody (1:1000 dilution), JAK2 antibody (1:1000 dilution) (Cell Signaling Technologies, Danvers, MA, USA), GAPDH antibody (1:2500 dilution), and IRDye 800CW or 680 LT secondary antibodies (1:1000).

### 2.5. FACS Analysis

Apoptosis of liver cancer cells treated with *Y-A* was determined by flow cytometry. LM-3 and HepG2 cells were seeded in 6-well plates at a density of 1 × 10^5^ cells per well 24 h before treatment with *Y-A* at concentrations of 0.1 μM, 0.3 μM, and 1 μM for 24 h. Cells were then stained with propidium iodide/Annexin V-FITC kit and apoptotic rates were determined by flow cytometry using FACSymphony™ A5 instrument.

### 2.6. Scratch Assays

HepG2 cells were seeded at a density of 1 million cells per well in 6-well plates. Following an overnight incubation period, a scratch was created using a 200-μL pipette tip. The cells were subsequently rinsed three times with phosphate-buffered saline (PBS) and imaged at the 0-h time point to establish a baseline. Cells were then treated with different concentrations of *Y-A* and incubated for 24 h before being photographed again. Wound healing was evaluated by comparing the migration of cells into the scratched area between the treated and control groups. Differences were analyzed using a *t*-test, with *p* < 0.05 considered statistically significant.

### 2.7. Transwell Migration and Invasion Assays

HepG2 cells were resuspended in serum-free DMEM and counted. A 24-well plate was prepared with 700 μL of DMEM containing 10% FBS in each well. A total of 20,000 cells were placed in the upper chamber of a transwell with serum-free DMEM. After 24 h, the chamber was fixed with paraformaldehyde, stained with crystal violet, washed with PBS, and observed under a microscope.

### 2.8. RNA Sequencing (RNA-Seq) and Docking Simulations

HepG2 cell lines were treated with 0.3 μM Y-A for 24 h before the cells were collected for RNA sequencing. The RNA was then extracted and subjected to sequencing using the Illumina platform.

The molecular structure of *Y-A* was retrieved from the PubChem compound database accessed on 5 July 2023 (https://pubchem.ncbi.nlm.nih.gov/), while the 3D coordinates of STAT3 (PDB ID: 3CWG, X-ray diffraction 3.05 Å) were obtained from the Protein Data Bank (PDB) (http://www.rcsb.org/). Docking was performed using a 30 Å × 30 Å × 30 Å cubic pocket with a grid spacing of 0.05 nm. Molecular docking simulation was carried out using AutoDock Vina 1.2.2 (http://autodock.scripps.edu/).

### 2.9. ELISA

To detect TNF-α in cell culture supernatants, HepG2 cells (5 × 10^5^) were treated with 0.3 μM *Y-A* for 24 h. After the treatment, the supernatants were collected and analyzed for TNF-α levels using a TNF-α ELISA kit (Abcam, ab181421, Cambridge, UK) according to the manufacturer’s instructions. For the detection of TNF-α in mouse serum, mice were first established with a liver cancer model. The mice were then treated with intraperitoneal injections of *Y-A* (2 mg/kg every two days) for two weeks. Subsequently, serum was separated and analyzed for TNF-α levels using a TNF-α ELISA kit (Abcam, ab181421) following the manufacturer’s protocol.

To detect IL-6 in cell culture supernatants, HepG2 cells (5 × 10^5^) were treated with 0.3 μM *Y-A* for 24 h. After this treatment, the cells were stimulated with 25 ng/mL TNF-α for two hours. The supernatants were then collected and analyzed for IL-6 levels using an IL-6 ELISA kit (Abcam, ab46027) according to the manufacturer’s instructions. For the detection of IL-6 in mouse serum, mice were first established with a liver cancer model. The mice were then treated with intraperitoneal injections of *Y-A* (2 mg/kg every two days) for two weeks. Serum was then separated and analyzed for IL-6 levels using an IL-6 ELISA kit (Abcam, ab46027) following the manufacturer’s protocol.

### 2.10. Nuclear Morphology Staining

To assess nuclear morphology, HepG2 and LM3 cells (5 × 10^5^ cells/well) were treated with various concentrations of *Y-A* (0.1 μM, 0.3 μM, and 1 μM) for 24 h. After treatment, the cells were stained with Hoechst 33,342 to visualize the nuclei. The nuclear morphology was then observed and recorded using a fluorescence microscope (Zeiss LSM780, Carl Zeiss AG, Oberkochen, Germany).

### 2.11. Statistical Analysis

ANOVA with Dunnett post hoc *t*-tests were used to compare differences between more than two groups. The data were presented as mean ± SEM. Other statistical analyses were analyzed using a Student’s *t*-test with GraphPad Prism 9 (GraphPad Software, New York, NY, USA). Statistical significance was set at **** *p* < 0.0001, *** *p* < 0.001, ** *p* < 0.01, *p* < 0.05.

## 3. Results

### 3.1. Cytotoxicity of Y-A Increases Dose-Dependently in Cancer Cells

**Figure 1:**
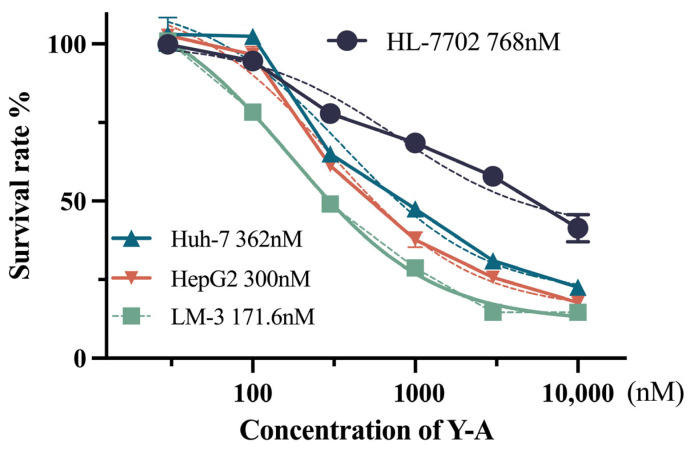
Effect of *Y-A* on cell viability in HL-7702, HepG2, LM-3, and Huh-7 cells. Cells were incubated with *Y-A* as indicated for 24 h. The IC50 was measured by GraphPad Prism 9 (GraphPad Software).

A CCK-8 assay was employed to evaluate the inhibitory effects of various concentrations of *Y-A* on tumor cell proliferation. As shown in Figure 1, two out of three liver tumor cell lines (HepG2 and Huh-7) did not exhibit significant proliferation inhibition at low concentrations (30 nM and 100 nM) of *Y-A*. However, approximately 20% inhibition was observed in LM-3 cells at 100 nM. With increasing concentrations of *Y-A*, cell viability in all tumor cell lines was dramatically suppressed. These results demonstrate that *Y-A* effectively inhibits liver tumor cell proliferation in vitro and suggest an optimal concentration range of 0.1–1 μM. The IC50 values for HepG2, Huh-7, and LM-3 cells were 300 nM, 362 nM, and 171 nM, respectively. In contrast, the IC50 for the normal liver cell line HL-7702 was 768 nM, indicating a higher tolerance to *Y-A* compared to the tumor cell line.

### 3.2. Y-A Treatment Ameliorates Migration and Invasion in Liver Cancer Cells

**Figure 2:**
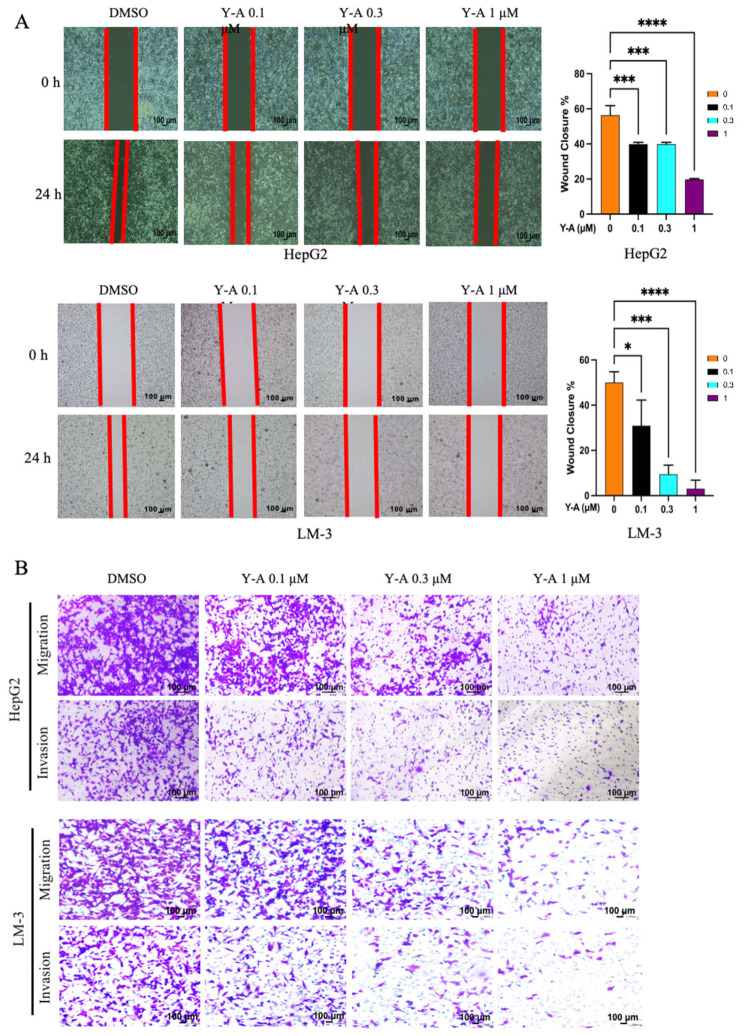
Treatment of *Y-A* ameliorates migration and invasion in HepG2 and LM-3 cells. (**A**) Migratory ability of HepG2 and LM-3 cells detected using scratch assay with and without *Y-A* treatment at indicated concentrations. Cells were treated for 0 and 24 h. (**B**) Transwell assay used to evaluate migration and invasion abilities of HepG2 and LM-3 cells with and without *Y-A* treatment at indicated concentrations. **** *p* < 0.000, *** *p* < 0.001, * *p* < 0.05. All values are expressed as mean ± SEM.

In order to examine the biological role of *Y-A* in hepatocellular carcinoma (HCC) cells, experiments including wound healing and transwell migration/invasion assays were performed. HepG2 and LM-3 cells were exposed to different concentrations of *Y-A* (0.1 μM, 0.3 μM, 1 μM). The scratch test results show a concentration-dependent inhibition of the wound-healing ability of HepG2 and LM-3 cells after *Y-A* treatment (Figure 2A). Furthermore, transwell assays revealed significant reductions in the migration and invasion abilities of HepG2 and LM-3 cells following *Y-A* treatment (Figure 2B). These results indicate that *Y-A* effectively inhibits the migration and invasion of HCC cells.

### 3.3. Y-A Induces Apoptosis in Liver Cancer Cells

**Figure 3:**
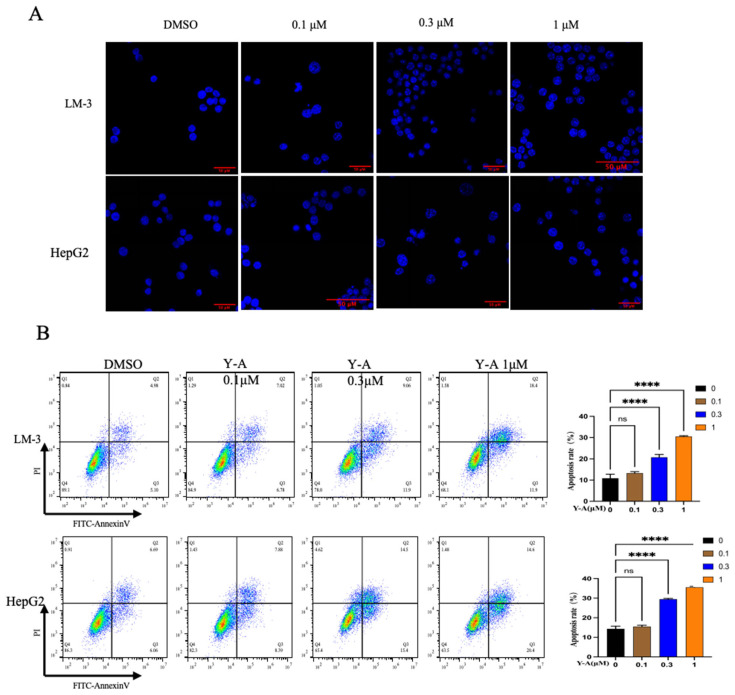
*Y-A* induces cell apoptosis in LM-3 and HepG2 cells (**A**) Hoechst 33,342 staining to detect apoptotic morphology in LM-3 and HepG2 cells treated with *Y-A* at indicated concentrations for 24 hours. (**B**) Representative flow-cytometric plots and quantification of percentage of apoptotic cells. LM-3 and HepG2 cells were treated with *Y-A* at indicated concentrations for 24 h, then stained with propidium iodide (PI) and Annexin V. **** *p* < 0.0001, All values are expressed as mean ± SEM.

The proliferation inhibition observed in the CCK-8 experiment suggests a need for further analysis of apoptosis. Higher concentrations of *Y-A* resulted in increased early and late apoptotic cells in both LM-3 and HepG2 cell lines (Figure 3A), consistent with the CCK-8 results. Interestingly, LM-3 cells exhibited more late (18.4%) than early apoptotic cells (11.9%); HepG2 cells exhibited the opposite trend, with a higher ratio of early (20.4%) compared to late apoptotic cells (14.6%). This differential response may be due to varying sensitivities to *Y-A*. Confocal microscopy confirmed these results, showing significant chromatin changes and increased apoptosome formation in treated cells (Figure 3B). These findings support the hypothesis that *Y-A* inhibits tumor cell proliferation and activates apoptotic signaling.

### 3.4. Y-A Inhibits Tumor Growth in Mice

**Figure 4:**
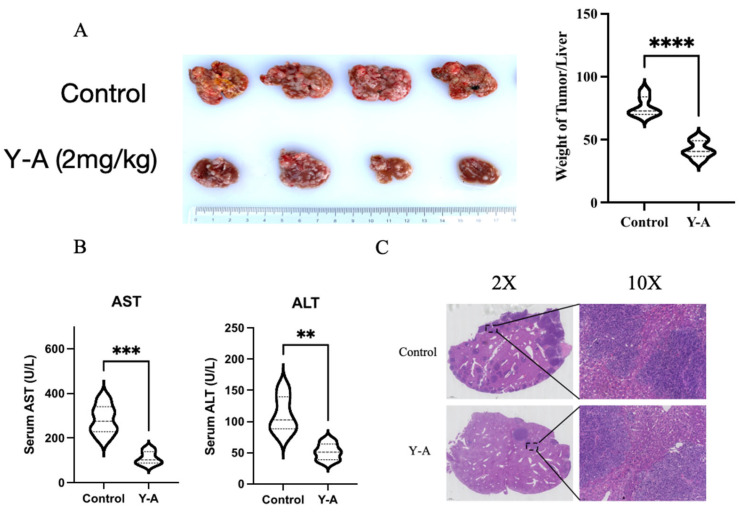
Y-A inhibits the growth of transplanted tumors in mice *(n* ≥ 5 per group). (**A**) After two weeks of Y-A treatment (2 mg/kg/day), the mice were euthanized and the liver tissues were extracted, photographed, and weighed as indicated. Y-A treatment group showed significant tumor growth inhibition compared to control. (**B**) Serum biochemical detection of ALT and AST protein levels after two weeks of Y-A treatment. (**C**) Representative H&E staining analysis of liver tumor lesions from mice. ** *p* < 0.01; *** *p* < 0.001; ****p < 0.0001, by unpaired Student’s *t*-test.

We employed an orthotopic liver cancer model using Hepa1-6 cells to assess the anti-tumor activity of *Y-A* in vivo. Mice received intraperitoneal injections of *Y-A* at 2 mg/kg/day for two weeks (Figure 4A). The *Y-A* treatment group exhibited a noteworthy suppression of tumor growth in comparison to the control group. Additionally, serum levels normalized with *Y-A* treatment (Figure 4B). Pathological analysis revealed significant reductions in liver tumor lesions in the *Y-A*-treated group (Figure 4C). These results indicate that *Y-A* effectively inhibits tumor growth in mice.

### 3.5. Y-A Targets the TNF-α/STAT3 Pathway to Mitigate Liver Cancer Progression

**Figure 5:**
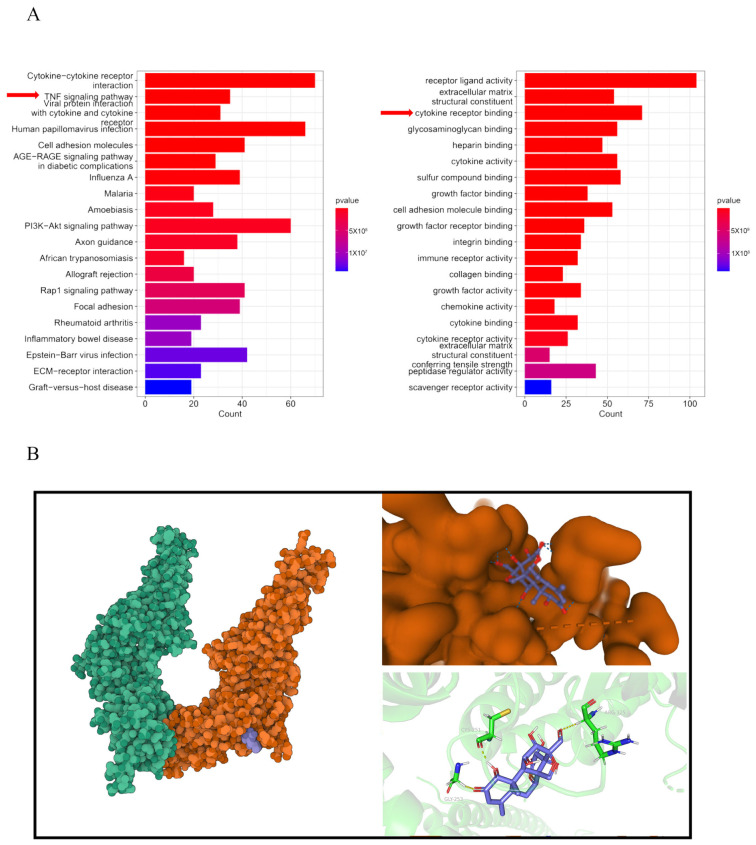
Y-A targets TNF-α/STAT3 pathway to mitigate liver cancer progression. (**A**) RNA sequencing (RNA-Seq) analysis was performed on cell lines treated with/without Y-A. KEGG pathway analysis and GO enrichment analysis performed as indicated. (**B**) Left: crystal structure of Y-A with superimposed features of STAT3 protein; right: interaction of the compound with its target and 3D structure of binding pocket. To provide a more detailed understanding of the molecular interactions, residues of Y-A binding locations are provided on STAT3.

**Figure 6:**
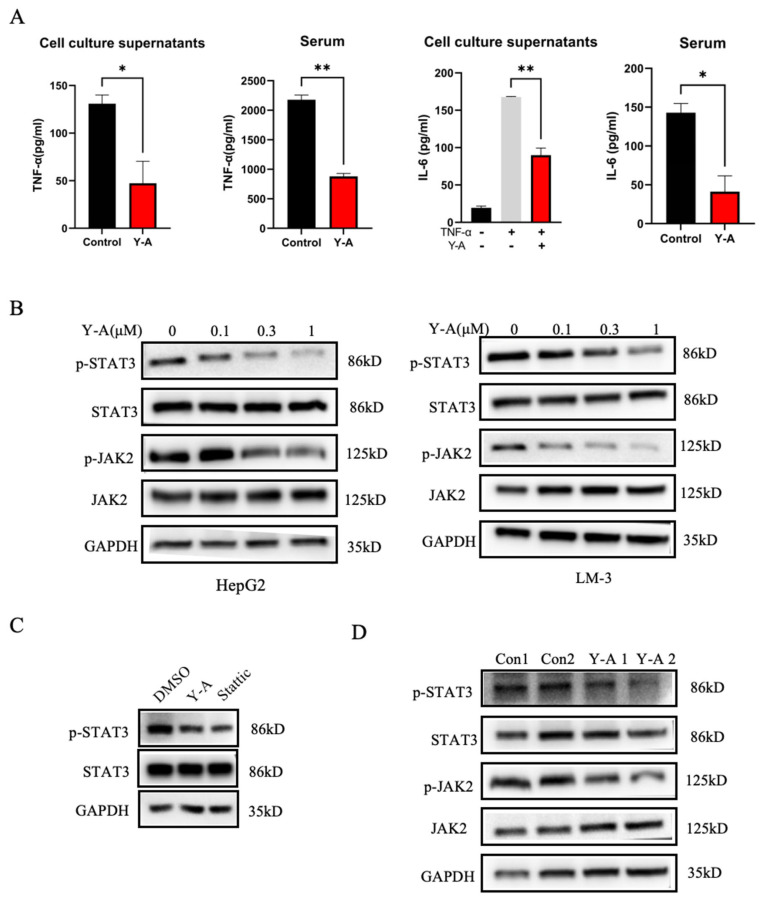
Y-A inhibits growth of liver cancer cells through JAK-STAT pathway. Representative Western blot shown. (**A**) Elisa detects expression levels of TNF-α and IL6 in cell culture supernatant and mouse serum, respectively. * *p* < 0.05; ** *p* < 0.01; by unpaired Student’s *t*-test. (**B**) Western blot analysis of STAT3, p-STAT3, JAK, and p-JAK in HepG2 and LM-3 cells. (**C**) Western blotting to detect effect of Stattic (5 μM), a STAT3 inhibitor, and Y-A on the phosphorylation of STAT3 and JAK in HepG2 cells. (**D**) Western blotting patterns of p-STAT3, STAT3, p-JAK2, and JAK from Y-A treated in tumor show decreased p-STAT3 and p-JAK2.

Treated HepG2 cell lines with 0.3 μM *Y-A* for 24 h before collecting the cells for RNA sequencing. RNA was then extracted and subjected to sequencing using the Illumina platform. RNA sequencing analysis revealed significant enrichment of the TNF-α signaling pathway in liver cancer cell lines treated with *Y-A* (Figure 5A). Chronic inflammation, a known risk factor for liver cancer [16], was supported by elevated IL-6 and TNF-α protein levels in mouse serum (Figure 6A). SuperPred analysis identified STAT3 as a potential target of *Y-A*. Molecular docking using AutoDock Vina showed that *Y-A* binds to STAT3 with a binding energy of −7.06 kcal/mol, indicating stable interactions (Figure 5B). ELISA assays confirmed a significant reduction in TNF-α expression after *Y-A* treatment (Figure 6A). These findings suggest that *Y-A* mitigates liver cancer progression by inhibiting the TNF-α/JAK/STAT3 signaling pathway.

### 3.6. Y-A Inhibits Liver Cancer Cell Growth via the JAK-STAT Pathway

In order to delve deeper into the impact of *Y-A* on the JAK-STAT signaling pathway, we conducted an analysis of STAT3 and JAK2 protein phosphorylation levels. The treatment with *Y-A* demonstrated a notable inhibition of STAT3 and JAK2 phosphorylation in a manner that was dependent on the concentration used (Figure 6B). Treatment with the STAT3 inhibitor Stattic also reduced STAT3 phosphorylation (Figure 6C). Consistent with in vitro findings, *Y-A* reduced phosphorylated STAT3 protein levels in liver tumor tissues (Figure 6D). These data suggest that *Y-A* inhibits HCC by targeting the STAT3 signaling pathway.

### 3.7. Y-A Induces Tumor Cell Apoptosis through the JAK/STAT3 Pathway

**Figure 7:**
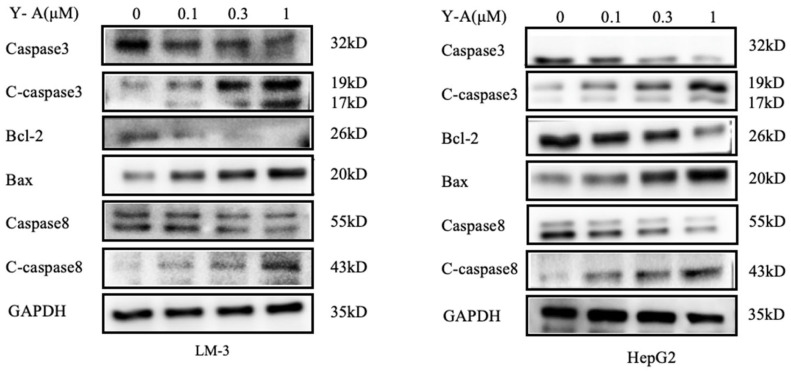
Y-A induces LM-3 and HepG2 cell apoptosis through JAK/STAT signaling. Y-A treatment inhibits STAT3, anti-apoptotic pathways, and disrupts Bcl-2 proteins. Western blotting patterns of Caspas3, Caspas8, Bcl-2, and Bax from Y-A treated in tumor showed that Caspase-3, Caspase-8, and Bcl-2 were decreased.

Studies have shown that STAT3 is overactive in many cancers. Inhibition of STAT3 can induce cell apoptosis and is closely related to key processes such as tumor proliferation, survival, and metastasis [17]. By inhibiting STAT3, anti-apoptotic pathways, including those regulated by Bcl-2 proteins, are disrupted [16]. Y-A treatment increased the levels of cleaved Caspase-3 and Caspase-8 in LM-3 and HepG2 cells, correlating with increased doses (Figure 7). Caspase-8 cleavage indicated activation of the extrinsic apoptotic pathway, while changes in Bcl-2 and Bax levels suggested activation of the intrinsic pathway. This indicates that Y-A induces apoptosis via the JAK/STAT3 pathway, where Caspase-8 activation leads to downstream Caspase-3-mediated cell death.

## 4. Discussion

Liver cancer is a significant health threat, particularly in China, where liver disease is prevalent [18,19,20]. Despite common treatments like radiotherapy, chemotherapy, and immune checkpoint inhibitors, recurrence rates remain high due to tumor heterogeneity and adverse effects. Plant compounds have shown promise as alternative cancer therapies due to their efficacy and safety [21,22].

This study investigated the anticancer activity of *Y-A* against liver tumor cells in vitro. A CCK-8 assay demonstrated a dose-dependent inhibition of cell viability in HepG2, LM-3, and Huh-7 cells. Additionally, *Y-A* significantly reduced the migration and invasion capabilities of HepG2 cells, indicating its potential as an effective HCC inhibitor. To assess the antitumor activity of *Y-A* in vivo, an orthotopic liver cancer model using Hepa1-6 cells was employed. The *Y-A* treatment group had a significant reduction in tumor growth compared to the control group. The findings of the study indicate that *Yadanziolide A* (*Y-A*) may have potential as a therapeutic agent for the treatment of hepatocellular carcinoma (HCC).

Apoptosis and autophagy are crucial mechanisms for eliminating proliferating cells and maintaining cellular homeostasis [23,24]. Understanding these processes is essential for developing cancer therapies [25,26,27]. This study demonstrates that *Y-A* induces apoptosis in HCC cells, as evidenced by significant nuclear fragmentation and changes in levels of proteins involved in cell death, such as Caspase-3, Caspase-8, Bax, and Bcl-2.

STAT3, often excessively activated in cancer, plays a crucial role in the signaling pathways of tumor cells. and immune interactions within the tumor microenvironment [28,29]. Targeting STAT3 offers therapeutic advantages by suppressing tumor proliferation and enhancing anti-tumor immune responses [30,31]. We found that *Y-A* inhibits STAT3 and JAK phosphorylation in a concentration-dependent manner, supporting its potential as an anticancer agent by modulating the STAT3 pathway. The study provides insights into the mechanism through which *Y-A* exerts its anticancer effects. This could pave the way for the development of targeted therapies that specifically modulate the JAK-STAT pathway in HCC.

Previous studies on alternative plant-derived compounds have shown varying degrees of success in inhibiting HCC progression [32,33]. For example, curcumin, derived from turmeric, has been extensively studied for its anticancer properties, including its ability to induce apoptosis and inhibit metastasis in HCC cells [34,35]. Similarly, resveratrol, found in grapes, has demonstrated potential in inhibiting STAT3 activation and inducing cell cycle arrest in various cancer cell lines [33,36,37]. However, the efficacy of these compounds often varies based on their bioavailability and the specific pathways they target.

In comparison, our study on *Y-A* shows a more pronounced inhibition of STAT3 and JAK phosphorylation, which suggests a potentially stronger effect on tumor cell proliferation and immune response modulation [33,34]. Additionally, while other plant-derived compounds have shown efficacy in vitro, *Y-A* has demonstrated significant tumor growth reduction in an in vivo orthotopic liver cancer model, highlighting its potential for clinical application.

Given the high recurrence rates and adverse effects associated with conventional HCC treatments like chemotherapy and radiotherapy [38], *Y-A* offers a potentially safer and effective alternative, leveraging plant-derived compounds for cancer therapy. However, the molecular mechanism underlying *Y-A’*s anticancer effect via STAT3 modulation requires further investigation. Future research should focus on the potential synergistic effects of *Y-A* when used in combination with existing treatments such as chemotherapy, radiotherapy, or immune checkpoint inhibitors to enhance overall treatment efficacy [39,40]. Additionally, a detailed exploration of the molecular mechanisms underlying *Y-A’*s inhibition of the JAK-STAT pathway and its impact on other signaling pathways involved in cancer progression and metastasis is needed.

## 5. Conclusions

In conclusion, *Y-A* exerts anti-tumor effects by inducing apoptosis and inhibiting STAT3 signaling in HepG2 cells and LM-3 cells, both in vitro and in vivo. The findings highlight that *Y-A* is a promising plant medicine that can be used in the treatment of HCC.

## Data Availability

The data of this study can be made available from the corresponding author upon request.
